# Match Analysis, Physical Training, Risk of Injury and Rehabilitation in Padel: Overview of the Literature

**DOI:** 10.3390/ijerph19074153

**Published:** 2022-03-31

**Authors:** Andrea Demeco, Alessandro de Sire, Nicola Marotta, Riccardo Spanò, Lorenzo Lippi, Arrigo Palumbo, Teresa Iona, Vera Gramigna, Stefano Palermi, Massimiliano Leigheb, Marco Invernizzi, Antonio Ammendolia

**Affiliations:** 1 S. Anna Institute, Via Siris, 11, 88900 Crotone, Italy; andreademeco@hotmail.it; 2 Department of Medical and Surgical Sciences, University of Catanzaro “Magna Graecia”, 88100 Catanzaro, Italy; nicola.marotta@unicz.it (N.M.); riccardo.span@gmail.com (R.S.); info@arrigopalumbo.com (A.P.); iona@unicz.it (T.I.); ammendolia@unicz.it (A.A.); 3 Physical and Rehabilitative Medicine, Department of Health Sciences, University of Eastern Piedmont, 28100 Novara, Italy; lorenzolippi.mt@gmail.com (L.L.); marco.invernizzi@med.uniupo.it (M.I.); 4 Neuroscience Research Center, Magna Græcia University, 88100 Catanzaro, Italy; gramigna@unicz.it; 5 Department of Public Health, University Federico II of Naples, 80131 Naples, Italy; stefano.palermi@unina.it; 6 Orthopaedics and Traumatology Unit, “Maggiore della Carità” Hospital, Department of Health Sciences, University of Eastern Piedmont, 28100 Novara, Italy; massimiliano.leigheb@med.uniupo.it; 7 Translational Medicine, Dipartimento Attività Integrate Ricerca e Innovazione (DAIRI), Azienda Ospedaliera Nazionale SS. Antonio e Biagio e Cesare Arrigo Alessandria, 15121 Alessandria, Italy

**Keywords:** padel, sports, sport injuries, rehabilitation, physical fitness, match analysis

## Abstract

Padel is a racket sport that has been gaining great popularity and scientific interest in recent years. It could be considered to be a high-intensity intermittent sport with valuable cardiovascular and neuromuscular benefits; however, the risk of injury cannot be neglected. To date, there is still a gap of knowledge in the scientific literature on this emergent sport. Therefore, the present review aims to synthetize the current knowledge on padel game dynamics to better characterize the main risk factors, the injury rate and characteristics, and the most effective rehabilitative treatment strategies. PubMed, Scopus, Cochrane, and PEDro were screened up to January 2022 to identify eligible studies focusing on padel players as participants. Out of 160 records, we included 19 studies, which were focused on match analysis, anthropometric and physical training, the risk of injury, and rehabilitative interventions. The results showed that the high action velocity and the sudden changes in direction during a padel match could represent a risk factor for injuries, especially in untrained players. However, the high heterogeneity of the studies in the literature hinders our ability to draw any strong conclusions, and the results should be carefully considered. Future research should address the lack of knowledge on injury mechanisms and type to implement a tailored rehabilitation program.

## 1. Introduction

Padel is a racket sport played in a grass court of 20 × 10 m, divided by a central net, and delimited by glass and gratings where the ball bounces on [1]. It has a Mexican origin (1969), adapting a squash court with elements of tennis [2]. Recently, an increasing number of players and courts have emerged in more than 40 countries [3]. Although similar to tennis, a padel court is about a third smaller than a tennis court, composed of a plexiglass wall at both end zones and completed by a metal mesh, which not only prevents the rise, but also reduces the effective playing area, and two openings at the outer side of the net; the padel ball also provides less bounce than a tennis ball [4]. Additionally, while tennis uses a stringed racket with a longer handle, padel provides a short-handled pad (racket) that has a foam core and a fiberglass or carbon fiber outer shell. Furthermore, in padel, the serve is performed by bouncing the ball and then hitting it while it is lower than the height of the hip, which is a less technical gesture than in tennis [5]. Finally, padel was conceived from the beginning as a doubles game, unlike tennis [6].

In this scenario, the key to this success is that high levels of technical ability could not be considered to be essential to begin practicing, it is usually played outdoors, and its equipment is inexpensive [7].

Throughout the game, similarly to other sports, players continuously solve their problems, collaborating to complete individual and cooperative activities to disturb the opponents and protect their side [8]. Therefore, the performance is the result of technical, tactical, and mental abilities. Thus, data on rally lengths might improve our understanding of match dynamics and athletes’ tactics and performance [9]. 

Moreover, this growth has also been reflected in an increase in scientific research, considering that half of the total number of the articles have been published in the last year. Existing literature addressed several research topics, such as athletes’ actions and distance covered during a game, match dynamics, the risk of injury, and physical fitness [7]. 

Padel has been defined as a high-intensity intermittent sport, combining high-frequency and low-intensity athletic gesture [10]. Apart from the dynamics of the game and the technical–tactical requirements, padel performances are influenced by the physical fitness and kinematic patterns of the athletes, especially in non-professional and recreational contexts with less demanding competitive requirements, but which can hide the risk of injury (e.g., microtrauma and overload disorders) [11].

**Figure 1:**
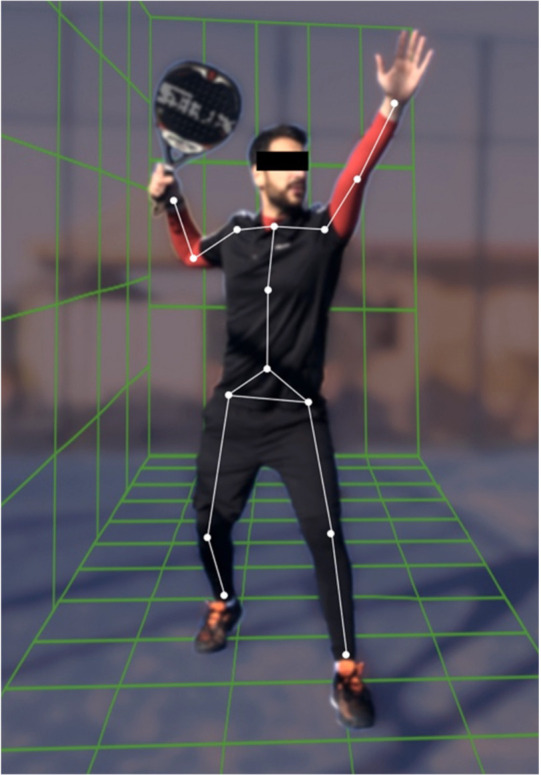
Smash is one of the most used and effective overhead strokes.

Knowledge of the loads of competition within the match would potentially improve the approach to the sports [12,13]. However, to the best of our knowledge, there is very little evidence on play patterns, athlete training, amateur–professional differences in performance, and the control of forces and energy expenditure, as well as the role of movement patterns and kinematics of the athletic gestures in padel players (see Figure 1). 

Furthermore, no previous reviews have investigated the impact of padel on the musculoskeletal system to provide clinically relevant data to guide physicians in effective and safe therapeutic strategies to prevent or manage sport-specific injuries. 

Therefore, the aim of this overview was to focus on the state-of-the-art research on padel in terms of match analysis, physical and anthropometric fitness, the risk of injury, and rehabilitation in players of this emerging sport. 

## 2. Materials and Methods

### 2.1. Data Search

Two authors examined four online databases: PubMed, Scopus, Cochrane, and PEDro. The articles’ selection was conducted throughout the search string: “Padel” (all fields). Furthermore, we analyzed the list of references of the papers included. The criteria utilized for the database analysis were: papers published up to January 2022; original articles, reviews, and commentaries; only articles in the English language. The selection was determined considering padel players as the study population. A third author solved the disagreements between the investigators regarding the study selections.

### 2.2. Data Extraction 

Data extraction was conducted following the Cochrane Review Group guidelines. We used an Excel sheet to evaluate inclusion requirements. The full text was analyzed, and the records were collected in the sheet. We extracted from the included articles the following data: authors; publication year; participant characteristics; design of studies; outcomes; main results.

### 2.3. Methodological Assessment

A modified version of the STROBE criteria [14] was utilized for conducting the methodological assessment. The papers were analyzed through ten criteria. Disagreements were examined and resolved by consensus. The items were assessed with a numerical categorization (assigning 1 if present, and 0 if non-present). The studies were classified with a high bias risk with a total score of <7; were considered to be low risk of bias if the score was >7. To conduct the clinical review, we considered previous guidelines, the research questions, appropriate evidence, the studies’ qualities, results synthesis, and their correct interpretation.

## 3. Results

### 3.1. Study Selection 

**Figure 2:**
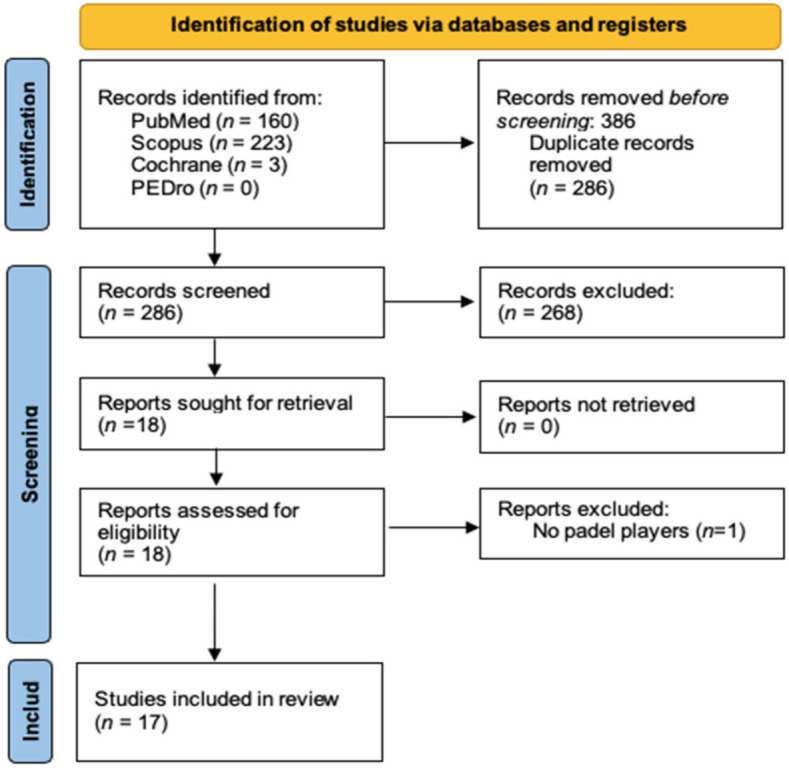
PRISMA flow diagram.

A total of 386 records were identified after the database search, which are detailed in the PRISMA flow diagram in Figure 2.

After duplication deletion, and the assessment of 386 studies for title and abstract, we considered 18 studies to be eligible. Lastly, one article was excluded for inconsistency with the eligibility criteria. As a result, 17 articles were elected for a deep analysis and included in the qualitative synthesis.

### 3.2. Main Characteristics of the Studies 

Table 1 summarizes the main characteristics of the studies. A total of 479 padel players with a different level of experience were included in the studies and 1098 matches were also analyzed. Most of the studies are cross-sectional (*n* = 3) [7,10,15] and observational (*n* = 12) [3,7,9,12,16,17,18,19,20,21,22,23]; studies on match analysis utilized the systematic observation of padel match videos from official channels. The current literature argues over four different aspects of padel: match analysis, anthropometric and physical fitness, the biological effects of padel matches on athletes, and the risk of injury and rehabilitative approaches. Almost all the study included were from Spain (*n* = 16) [3,7,9,10,12,15,16,17,18,19,20,21,22,23,24,25]; only one was from Sweden [26]. Ten of the included studies investigated game dynamics [3,9,12,16,17,18,19,20,21], of which one [12] analyzed the court zone involved in the attacking and defensive phase of the match; six studies [3,16,17,19,20,21] investigated padel strokes, highlighting that the smash is one of the most effective strokes with which to stop the rally; on the other hand, the lob is the most valid in a defensive position [20]. 

**Figure 3:**
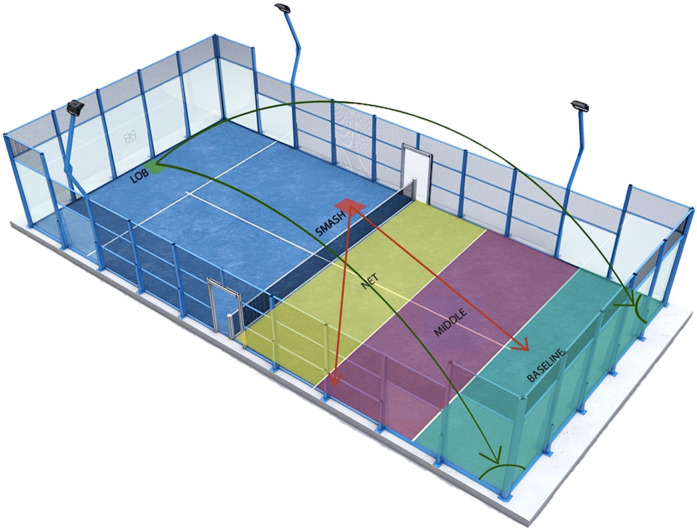
Game stroke strategies during a Padel match based on game dynamics.

However, the athletes during match prefer performing groundstrokes (15.48%) and volleys (25.09%) (see Figure 3).

Three studies [9,18,28] considered how the length of the rally changes based on experience [18] and the seconds from the serves [9]. The physical analysis conducted in two studies [10,15] revealed that padel players presented a healthy body composition and professional players had considerably lower levels of body fat and thigh fat area when compared to the general population, and significantly greater lumbar isometric strength. Padel athletes can be qualified as endo-mesomorphic [15]. Quesada et al. [24] examined the risk factors in padel players through a retrospective questionnaire, revealing that 40% of the players sustained on injury in the last year; in addition, the unpredictable trajectory and the velocity of the ball could be responsible for eye injuries [26]. As reported in three studies, a padel match has both biochemical and psychological effects. In detail, a simulated padel match caused a rise in brain-derived neurotrophic factor (BDNF) level from 1531.12 ± 269.09 to 1768.56 ± 410.75 ng/mL in female players [25]; moreover, there is an increase in urinary-specific gravity for male players, in contrast to female players, and a significant increase in microalbuminuria from 47.25 ± 18.81 to 207.51 ± 147.11 (g/L); furthermore, players self-reported a significant increase in mental fatigue after a padel game that worsened after the second consecutive match.

### 3.3. Methodological Quality 

Table 2 describes the studies’ methodological quality scores [29]. Eleven studies [7,10,15,16,17,19,21,22,23,25,28] had 10 points; three studies [9,18,24] had 9 points, two studies had 8 points [12,20], and one [26] had 5 points. 

## 4. Discussion

The aim of this comprehensive review was to provide an overview of match analysis, physical and anthropometric fitness, injury risk, and rehabilitation approach in padel players. Unfortunately, little evidence is present in the literature, and the few studies reviewed provide heterogeneous results in terms of injury mechanism descriptions, injury rates, and rehabilitation interventions. However, we found promising considerations regarding match analysis and kinematic evaluation. Considering the growing number of paddle players in the world and its rapid spread in different countries, this review could be a starting point to promote better knowledge of the mechanisms underpinning padel-related injuries to implement both preventive and rehabilitative strategies.

### 4.1. Match Analysis

#### 4.1.1. Court Zone

The court is a rectangle 10 m wide by 20 m long (interior measurements) with a 0.5% tolerance [4]. The region of the court (net, middle, and baseline) greatly influences the dynamics of the game. Herein, 46.6% of the activity takes place in the baseline area, while the 27.7% occurs in the middle area and 25.6% near the net. At the baseline, side corners gain great importance, whereas the center is significant in the middle and net part of the court [9].

Most of the winning points are scored from the middle and net region of the court (respectively, 76.3% and 41.2%) through two strategies. The first involves sending the ball to the side-corners, at the baseline near the walls, through lobs, keeping the opponents away from the offensive areas; the second is to use volleys to protect the central space between players and to prevent deep balls that could cause the loss of the advantage position. Therefore, in the defense phase, the baseline corners acquire greater importance, while the central line is crucial when attacking (25% of the game) [9].

The net position significantly increases the efficiency of winner points thanks to the advantageous position of the attackers (low error rates), and a minor time of reaction for the defending couple [17]. There is a continuous movement of the players, whose purpose is to conquer the position near the net, which is considered to be more advantageous, whilst the defensive pair attempt to recuperate it [17,30].

In addition, the strategy to approach the net in the central region has two aims: to protect the court between players and to control a larger angle, increasing the possibilities of a winning point; consequently, the capacity of volleying in the central region lets athletes spend more time near the net, increasing the probability of scoring [9].

#### 4.1.2. Padel Strokes

Volleys, serves, and groundstrokes are the most frequent strokes [16]. The type of shot depends on the phase of the game, in particular, whether one is in a defensive position, wherein lob or passing shot are performed, with variable height and directions, in order to displace the attacking pair [17,20]; the most efficacious strokes during a game are the overhead strokes (smash and tray); in particular, 8.6–7.2% of trays and 33.3–51.8% of flat smashes are winning shots [17]. The smash has a great influence on the outcome of the game and its success is related to the court area, direction, and the speed and precision with which it is performed [17]. 

Escudero-Tena et al. [20] reported that the lob is the most frequent (85.4%) and effective shot in a defensive position, especially for women, to overcome the position of the opponents at the net. Though it is not a closing shot, it allows the continuity of the game, increasing the length of the rally. Therefore, in unfavorable situations, the lob is the recommended shot to contrast the opponent’s attacks, gain the net, and obtain an advantage in the rally [20].

Ramón-Llin et al. [21] focused on the importance of two aspects of padel strokes: direction and effectiveness. The use of cross-court shots bounces the ball on the railing or wall, increasing opponent’s uncertainty and the possibility of making a mistake. 

Focusing on the effectiveness of a stroke, the winning couple presented a greater percentage of break points won and a lower percentage of errors (7.5%) [3]. In detail, the winning players executed a higher number (85% of the score) of attacking strokes in a game, executed with flat hits and topspin [3].

Even the experience influences the type of strokes in a padel match, with beginners utilizing, as a percentage, more stroke-like serves (21.46), groundstrokes (23.98), and lobs (11.40), and professional players utilizing volley (25.09%), wall strokes (22.85%), and smashes (4.84%) [16], probably due to the particular attention of an experienced athlete on their positioning and movement approaching the net, and during the time spent at the net. 

Usually, the left player takes more hits than the right player, playing more offensively. This is due to the fact that in right-hand-dominant couples, the left-side player performs the stronger smash, as they can execute the stroke with the dominant limb in the center of the court. However, a left-hander on the right part of the court defends the center line more effectively [31]. 

#### 4.1.3. Rally

The length of time between the serve and the point is called a rally. The length of the rally is related to the experience of the players. In addition, winning pairs play longer (+20%) rallies. Overall, most points are scored between the fifth and eleventh second of the rally. The probability of making an error following an unforced hit is maximum (40%) in the first 4 s of the rally; on the other hand, the chance of an error following a forced hit increases after the eleventh second of the rally (30%). Therefore, the ability of an elite player lies in avoiding unforced errors in the first seconds of playing the rally [9].

As regards the male and female padel players in the Under 16 (U16) and Under 18 (U18) groups of the 2014 Spanish National Youth Championship, the activity of padel matches in young people was characterized by a longer duration of rallies, more shots per rally, and longer rest times than other racquet sports [18]. Specifically, the main results showed that U18 athletes, in relation to U16s, showed better physical conditions and higher technical qualities. The increase in age and experience is correlated, in both women and men, with an increase in the effort index and, consequently, in the physiological needs during physical activity, suggesting that balance and response time are fundamental characteristics of the elite player. Moreover special attention should be given to physical padel training, as well as psychological skills and execution behavior [9].

### 4.2. Physical Fitness

Body dynamics and anthropometric characteristics are linked with higher results in several sports, but most importantly in racket sports [10,32]. 

The height and arm span of a player are crucial, and high power is essential to complete the exchanges, taking advantage of the most powerful shots, which are those performed overhead [12]. In this context, taller padel players with higher muscle weights could execute very powerful strokes (which can sometimes take the ball out of the court, scoring a direct point) [10]. Height seems to be crucial in the effectiveness of a smash. Male players show a significantly higher height compared with female players (1.80 ± 0.06 m vs. 1.67 ± 0.06) [33].

Padel athletes usually shift their stance quickly and require high leg power to shift upper body weight quickly. For example, mid-range smashing skills (which occur in less than 10% of the match, but which are performed at high velocity) or volleys near the net (which occur in more than 30% of the match) are essential to increase the score. Consequently, an higher “core” strength is essential to execute potent shots [10].

Moreover, the presence of walls and gratings that surround the field that can be hit by the ball extends rally duration, and, therefore, the amount of actions and strokes is greater than other racket sports, such as badminton and tennis [9]. For these reasons, padel is considered to be an intermittent high-intensity sport, which alternates between high-frequency and low-intensity activities, interspersed with 1020 s rest in between [10,34].

While at non-professional levels, physical conditioning has low influence on the performance of the padel player, the increasing intensity of elite competitions makes strength and conditioning training a priority for success [10]. In more detail, a study conducted by Courel-Ibanez [35] on middle-aged adult women who play padel regularly reported an improved physical conditioning and body health compared with sedentary women, with a higher proprioception, strength, and cardiorespiratory endurance. Furthermore, they reported minor abdomen, hip circumference, and leg skinfold differences to sedentary women, lowering the risk of cardiac disorders, osteopenia, and back pain [35].

Male professional padel players are shown to have high levels of cardiopulmonary capability, upper body and grip strength, and rapidity, highlighting the role of padel in improving favorable adaptations in the cardiovascular system similarly to other sports [36,37]. Conversely, the players presented a low value of dynamical stability in the rear and front directions [7]. 

In detail, professional athletes train about 23.5 h a week, have an average height of 177.9 (4.0) and weight of 78.2 (8.5), with a fat mass percentage of 12.53 (4.83)%; furthermore, they have a VO2 max (mL/kg/min) of 55.43 (7.04) and a hand dynamometer power of 51.14 for the dominant hand [7].

The values of fat mass are comparable to the results of other racquet sports, where the range obtained was between 12% and 15%, and the energy expenditure is similar for tennis [7]. Significant differences between the sexes are reported in approximately all fitness indexes, particularly for the arm, both in maximum dynamical and explosive power.

Moreover, male padel players have greater explosive power than women, in line with competition requirements, in which there are quicker rallies and more powerful shots (smash). Since individualization represents an essential principle in training, coaches should consider the cardiorespiratory, strength, and agility values to optimize preparation and test procedures for the athletes [7].

Considering young players, both sexes showed comparable performance in the analyses, except for jumps [15]. Young padel players have a BMI (kg/m^2^) of 22.6 (3.4), with a % of fat mass (kg) of 19.7 (7.2). The strength values of the upper and lower limbs are lower than in other racquet sports [15,38], which is probably caused by the playing actions that are different to other racquet sports [18]. On the other hand, even in young players, padel may play a key role in improving body composition and mitigating the worrying level of obesity in young people [15,39].

### 4.3. Biochemical and Psychological Effects of a Padel Match

The biochemical and psychological impact of padel on health status have been recently investigated by Pradas et al. [25], who suggest that there is a positive influence of padel on brain health. In more detail, they found a significant increase in BDNF in a blood sample taken after a simulated padel match. BDNF is a fiber-derived peptide (myokine) produced by skeletal muscles and regulated by physical exercise [40] that plays a key role in neuroplasticity and can also influence the regulation of muscle metabolism, enhancing glucose consumption and fat oxidation, highlighting the biological neuroprotective and regenerative properties of sport in the central and peripheric nervous system.

On the other hand, intensive padel training can cause neuromuscular fatigue in handgrip strength tests, especially in women [23]. However, the data reported highlight that a simulated padel match did not raise the levels of neuromuscular fatigue in the lower limbs thanks to frequent pauses, highlighting the importance of recovery in reducing fatigue in high-intensity intermittent sports, including padel [41]. 

Pradas et al. reported a modification in urinary values after a padel match; specifically, they found a worsening of the urine-specific gravity, which is considered to be a valuable index for sports people’s hydration status, particularly in men [23]. The gender difference found could be due to an higher percentage of hypohydration in men [42] and the tendency of less sweating of female players (due to anthropometric and metabolic characteristics). Moreover, the same study reported an increment in microalbuminuria excretion in both genders, a parameter that normally increases after intense physical exercise [43], and which is caused by a reduction of lumen in the kidney arteries, slowing renal plasma flow throughout intense activity [44]. 

Alongside this, it is equally important for elite athletes to investigate mental fatigue. This state was defined by Van Custem et al. [45] as a psychobiological impairment caused by significant mental efforts that require a high cognitive and emotional commitment. This aspect is particularly relevant in padel, considering the significant amount of cognitive effort in seeking appropriate solutions in an unpredictable and high-speed environment [9]. The use of railings and walls raises the entropy and the variability of the technical–strategic reactions related to the highest increases in mental fatigue. Díaz-García et al. [22] demonstrated an increase in mental fatigue specifically if there were more consecutive games, confirming how adequate recovery time remains a cornerstone for preventing both neuromuscular and mental fatigue. Motivation, which during exercise increases the contribution of the facilitation system (improving the capacity of the athlete to tolerate exertion and improving physical performances), might decrease after a padel match, with negative emotions caused by performance dissatisfaction. Even reaction time, which according to Le Mansec et al. [46] consists of a cognitive part (attentiveness to feel the stimulus) and a neuromuscular part (quicker reaction), is reduced after a game due to the psychophysical effort required during the game. Filipas et al. [47] suggest that a specific conditioning regime improved athletes’ mental fatigue endurance in sports, and the intake of caffeine and creatine could have a positive effect [48], improving the availability of the extracellular adenosine in the brain [49]. However, mental fatigue improves in the following days due to the positive intervention of rest or sleep [49].

### 4.4. Risk of Injury and Rehabilitative Strategies

There is still little evidence in the current literature on this topic. The largest contribution has been provided by the study of Priego Quesada et al. [24], who investigated the injury rate in a regular season and the features of the injuries in a population of recreational padel players. The authors found no clear correlations between gender, age, frequency of paddle sessions, and athletes’ experience with the risk of sustaining an injury. Furthermore, there are conflicting results regarding the association between padel shoes and injury risk, and the topic requires further examination.

Two out of five padel players have suffered at least one injury during the last year. Among non-professional players, 53.1% of all injuries required more than a month of recovery.

The majority of padel injuries target the lower limbs (53.1%), and sprains were the most common lesions, classically consequent to an abrupt internal inversion and rotation when the player executes a change in direction. Such movements occur with a high frequency in padel.

Moreover, padel players reported a large percentage of upper limb injuries (37.5%). Considering that padel commonly implies overhead strokes (e.g., smashes), the majority of the padel injuries affect the shoulder and elbow, causing rotator cuff syndrome, subacromial impingement, bursitis, and epicondylitis. The difference in the proportion of upper extremity injuries in padel and other racquet sports may be due to the court being smaller than in tennis, which can increase the frequency of shots taken. This potential for greater repetition of the abduction–external rotation movements of the arm during these strikes could explain the higher rate of upper extremity injuries particularly in case of shoulder instability [50,51]. Thus, it could be considered mandatory an early diagnosis of the kinematic alterations to plan a global rehabilitative approach to the injury [51,52]. 

In addition, a Swedish study points out that the small area of the court, the proximity of the players, the size and speed of the ball, and the risk of unpredictable ball bounces represent risk factors for eye injuries, and propose the use of protective eyewear to reduce the number of eye injuries [26]. 

Therefore, figures such as the coach or the athletic trainer should introduce a prevention program of exercises for avoiding performance decreases or a stop in training caused by a lesion, mainly when the player is approaching sport periods [12]. The smaller court size in padel, as well as the quicker pace of play, may also lead to faster and more intense gameplay, which may contribute to injury risk [52,53,54], mostly if fatigue is coupled with improper technique [41,55]. In addition, the high intensity of running and jumping may affect the ability to control landings, and may consequently be associated with non-contact injury [56]. Furthermore, excessive training or exercise load may also be associated with injury risk, highlighting the need to focus on proper technique, rest, and recovery [54,57]. 

Complementary postural exercises and trunk “core” strength seems to be essential for avoiding injuries with long-term consequences [35], since the unilateral overhead sport nature of padel means that athletes are specialized in playing in a specific part of the field, which has a strong influence on game movements [30,35]. 

In this context, pre-season screening plays a key role, with pre-season tests, both clinical and instrumental (e.g., surface electromyography and inertial measurement unit), that could identify athletes’ deficits (e.g., neuromuscular or movement deficit) to target them with a rehabilitative strategy [58,59].

In summary, two out of five padel players experienced an injury in the past year and most of the injuries affected the lower limb, wherein ankle sprains were the most common injuries [24]. Moreover, rotator cuff diseases, subacromial impingement, bursitis, and epicondylitis are also common due to the nature of overhead play. The difference in the proportion of upper limb injuries in padel and other racket sports may be due to the court being smaller than in tennis, which can increase the frequency of shots taken [24]. Furthermore, the proximity of the players, the size and speed of the ball, and the risk of unpredictable ball bounces are risk factors for eye injuries, suggesting the use of protective eyewear.

### 4.5. Study Limitations 

The present study is not free from limitations: firstly, we considered a small number of articles due to the low availability of scientific evidence; secondly, there is a lack of data on padel injuries and the correct rehabilitation strategy for these patients; lastly, considering that padel is a sport with a widespread practice in Latin American countries, we are aware that papers published only in the Spanish language have not been included in the present overview and could be considered for further studies. 

## 5. Conclusions

Taken together, the results of this literature overview revealed that the intermittent high-intensity physical activity and continuous neuromuscular stimuli during padel matches might have relevant cardiovascular and psychological benefits for padel players. A player’s height and arm span play a crucial role in padel; furthermore, players typically need to change positions quickly and require high leg power to shift their upper body weight quickly. In this sense, a more powerful “core” is essential to perform effective shots. The biochemical and psychological effects of padel on one’s health have been reported, with a significant increase in BDNF. On the other hand, padel requires a considerable amount of cognitive effort and appropriate solutions in an unpredictable and high-speed environment. Despite the low evidence on injuries, special attention should be given to the ankle, shoulder, and elbow.

However, we should underline the high degree of heterogeneity in the studies and outcomes in the literature, despite the popularity gained in the recent years by this sport. This issue should be adequately addressed, considering that padel, given its attractiveness, could be considered to be an important health promoter with relevant implications in terms of active lifestyle and global health implementation. Thus, future studies should focus on the mechanisms of injury, prevention programs, and rehabilitation strategies for a better management of padel players.

## Figures and Tables

**Table 1:** Main characteristics of the included studies.

Article	Nationality	Design	Participants	Aim	Procedure	Main Outcomes
Sánchez-Alcaraz et al., 2021 [16]	Spain	Systematic observation	Male padel players (*n* = 48; 24 trained and 24 novice).	To determine the differences in ball impact positions regarding nine stroke types	Type of stroke and ball impact position were registered using a computerized motion racking video system	Results showed that trained players hit the ball in a more backward position in serve and offensive shots but used more forward strokes in defensive shots.
Sánchez-Alcaraz et al., 2020 [17]	Spain	Systematic observation	Professional padel players (*n* = 20)	To analyze the distribution and effectiveness of the different types of smashes	Identification of the variables (type of smash, shot effectiveness, hitting zone, and shot direction) through use of observational instrument software	The tray was the most commonly used smash. Female players used more tray and fewer flat and topspin smashes than male players. Men executed a significantly higher percentage of winning smashes than women. Flat smashes and off the wall smashes were predominantly down the line strokes and women performed significantly more cross-court topspin smashes than men.
Courel-Ibáñez et al., 2019 [12]	Spain	Systematic observation	Male professional padel players (*n* = 4)	To assess emerging players’ responses within a natural competitive environment	Game dynamics were analyzed regarding the technical (groundstroke type, swing, and height), spatial (depth and laterality), and effectiveness indicators through Lince video analysis software	The results revealed three main game styles influenced by the court zone (net, middle, and baseline). In net and middle areas (offence) stood the use of volleys and overhead strokes on the center lane to keep a positional advantage and solve the point. Conversely, in the baseline (defense), the use of corner side walls and the domain of lobs showed to be relevant.
García-Benítez et al., 2018 [18]	Spain	Systematic observation	Young padel players (*n* = 32)	To assess match activity profile and temporal structure	The activity profile was determined by filming each match with two video cameras positioned 2 m from the back of the court	Young athletes playing longer rallies had a longer resting interval time resulting in a lower effort index. Results revealed an increment on match requirements in under 18 players compared with under 16 s.
Escudero-Tena et al., 2021 [3]	Spain	Observational study	1070 sets from 532 matches	To analyze performance indicators and their influence on match outcomes regarding sex, tournament round, and set number	The matches were downloaded from the official channel of the World Padel Tour and analyzed through Lince video analysis software	Winning couples showed a significantly higher number of break points won, total break points, smash winners, total smashes, total winners, volley winners, and significantly fewer errors in both men and women.
Ramón-Llin et al., 2021 [19]	Spain	Systematic observation	Padel players (*n* = 72)	To analyze the influence of service tactic formation on players’ movements and point outcome at two different performance levels	Two digital videocameras were used to film the matches; players and ball coordinates were analysed using a computerized motion tracking system (SAGIT/Squash)	Elite players had a significantly higher usgae share of the Australian formation compared with novices. Servers were significantly closer to the net and the side wall using a conventional formation when the returner hit the ball. Furthermore, servers had to move quicker when they used the Australian formation.
Courel-Ibáñez et al., 2017 [9]	Spain	Systematic observation	1527 rallies from 10 male matches	To analyze differences in rally length considering attack effectiveness, players’ locations and game outcomes	The matches were analyzed from the official YouTube channel of the World Padel Tour; data were collected through systematic observation	Winning pairs played longer rallies than losers. In detail, most winners’ errors were made at the net, while most points were scored at the baseline. Winners played longer rallies compared to losers, resulting in better performance.
Escudero-Tena et al., 2020 [20]	Spain	Observational study	Women players in the 2018 World Padel Tour (WPT) (*n* = 10)	To analyze game dynamics considering padel strokes	The instruments and materials used in the research were an “ad hoc” observation sheet; the LINCE software was used for video analysis [27]	The lob is the most frequently utilized and valid shot of women in the defensive position, who use it to pass the offensive position of the opponents.
Ramón-Llin et al., 2020 [21]	Spain	Systematic observation	Padel players (*n* = 24) performed 8441 shots from 9 matches	To analyze the distribution of padel strokes, their effectiveness, direction, and court zone	Two digital video cameras were used to film the matches; the data were recorded, using specific software for video analysis: LINCE software	Winning couples made a significantly higher number of winning and cross-court smashes and volleys from the offensive zone. Furthermore, players on the left side executed a higher percentage of cross-court and winning shots than the players on the right side.
Priego Quesada et al., 2018 [24]	Spain	Retrospective study	Recreational padel players (*n* = 80)	To examine the association between intrinsic/extrinsic factors and injury in recreational padel players.	Retrospective self-administered questionnaire	40.0% of the players suffered at least one injury during the past year. Sex, age, frequency of padel sessions/week, and years of padel experience were not correlated with injuries.
Thörnland et al., 2021 [26]	Sweden	Case reports	Padel players (*n* = 3)	To describe three cases of blunt eye trauma	Three cases of eye injuries during padel games were described and subsequently examined and treated surgically	The size and velocity of the ball, the risk of unpredictable bounces of the ball, and the relatively close distance between the players are important risk factors. The most effective method of reducing the amount of eye injuries is the use of protective eyewear.
Pradas–Sánchez-Pay A, et al., 2021 [7]	Spain	Cross-sectional study	Professional padel players (*n* = 30)	To examine the fitness characteristics of professional padel players and to determine differences in physical performance regarding players’ gender	The analysis was conducted on data collected during a national players’ meeting using a series of standard anthropometric and physical performance tests	Male players showed better values in terms of weight, height, one repetition maximum, jump test, and VO2 max test than the women. By contrast, the women had higher levels for fat mass. Males have higher explosive strength and more explosive shots than the females players.
Pradas–Cádiz et al., 2021 [25]	Spain	Observational study	Trained padel players (*n* = 24)	To evaluate the responses of brain-derived neurotrophic factor (BDNF), leukemia inhibitory factor (LIF), and irisin (IR) to padel competition in trained players	Circulating levels of BDNF, LIF, and IR were measured before and after simulated padel competition	The results suggest that competitive padel practice induces a slight but significant response of BDNF in female players. However, padel competition did not influence the release of LIF and IR.
Díaz-García et al., 2021 [22]	Spain	Observational study	Professional padel player (*n* = 14)	To quantify the evolution of mental fatigue, motivation and reaction time during a WPT competition	Mental fatigue and motivation and reaction time, with a 3 min Psychomotor Vigilance Test, were assessed at two time intervals	A rise in mental fatigue after the match was detected, with an accumulation of mental fatigue between matches played on day 1, maximizing the mental fatigue perceived during match 2.
Pradas et al., 2021 [23]	Spain	Observational study	High-level padel players (*n* = 28)	To evaluate neuromuscular, urinary, and hematological responses after simulated padel competition and analyze possible gender differences	Neuromuscular, hematological, and urinary parameters were analyzed before and after a simulated game	Significant gender differences were found in neuromuscular and hematological responses, with men showing higher values. After a simulated game, ABK and microalbuminuria levels changed. The impairment in hand grip strength, SJ, CMJ, and ABK was higher for men than women. The simulated game negatively affected the neuromuscular parameters.
Sánchez-Muñoz et al., 2020 [10]	Spain	Cross-sectional study	Male padel players (*n* = 60)	To describe and compare the anthropometric and physical fitness of male padel players according to their competitive level	Anthropometric variables, hand grip and lumbar isometric strength, flexibility, and lower-body muscular strength were analyzed	Elite athletes presented lower levels than subelite players for thigh and calf skinfolds. Elite players show significantly lower levels of body fat and thigh fat area, and significantly higher lumbar isometric strength. Endo-mesomorphic is the somatotype of the elite padel athletes.
Courel-Ibáñez et al., 2021 [15]	Spain	Cross-sectional study	Padel players (*n* = 34)	To examine the fitness characteristics and to identify the influence of gender and practice experience between young amateur padel players	Body composition was measured through bioimpedance; change in direction and agility were assessed by two padel-adapted tests	Male and female young padel players showed a healthy body composition and comparable results in all fitness analyses excepting for jumps.

**Table 2:** Methodological assessment of the included studies.

Articles	Criteria for the Quality Scoring										Score
	1	2	3	4	5	6	7	8	9	10	
Sánchez-Alcaraz et al., 2021	1	1	1	1	1	1	1	1	1	1	10
Sánchez-Alcaraz et al., 2020	1	1	1	1	1	1	1	1	1	1	10
Courel-Ibáñez et al., 2017	1	1	1	1	1	0	1	1	1	0	8
García-Benítez et al., 2017	1	1	1	1	1	1	1	1	1	0	9
Ramón-Llin et al., 2021	1	1	1	1	1	1	1	1	1	1	10
Courel-Ibáñez et al., 2017	1	1	1	1	1	1	1	1	1	0	9
Escudero-Tena et al., 2020	1	1	1	1	1	0	1	0	1	1	8
Escudero-Tena et al., 2021	1	1	1	1	1	1	1	1	1	1	10
Ramón-Llin et al., 2020	1	1	1	1	1	1	1	1	1	1	10
Priego Quesa et al., 2016	1	1	1	1	1	1	1	1	1	0	9
Thörnland et al., 2021	1	1	1	0	0	0	1	0	1	0	5
Pradas–Sánchez-Pay A et al., 2021	1	1	1	1	1	1	1	1	1	1	10
Pradas–Cádiz et al., 2021	1	1	1	1	1	1	1	1	1	1	10
Díaz-García et al., 2021	1	1	1	1	1	1	1	1	1	1	10
Pradas et al., 2021	1	1	1	1	1	1	1	1	1	1	10
Sánchez-Muñoz et al., 2020	1	1	1	1	1	1	1	1	1	1	10
Courel-Ibáñez et al., 2021	1	1	1	1	1	1	1	1	1	1	10

The abstract is informative and balanced (1); presence of specific objectives, including any prespecified hypotheses (2); presence of the eligibility criteria (3); for the variables of interest, presence of sources of data and characteristic of measurement methods, and description of equivalence of methods when there are two or more groups (4); explains if variables were quantitative (5); outlines characteristics of study population (6); highlights key results based on study aim (7); presence of limitations of the study (8); careful interpretation of results, reflecting objectives, similar articles, and other relevant evidence (9); funding statement (10).

## Data Availability

Not applicable.
